# Performance of ChatGPT-4 in answering questions from the Brazilian National Examination for Medical Degree Revalidation

**DOI:** 10.1590/1806-9282.20231362

**Published:** 2024-04-22

**Authors:** Somsri Wiwanitkit, Viroj Wiwanitkit

**Affiliations:** 1 Private Academic and Editorial Consultant – Bangkok, Thailand.; 2 Saveetha University, Saveetha Institute of Medical and Technical Sciences, Saveetha Medical College – Chennai, India.

Dear Editor,

We follow the topic entitled "Performance of ChatGPT-4 in answering questions from the Brazilian National Examination for Medical Degree Revalidation^1^." The purpose of this study was to evaluate ChatGPT-4's performance in answering the 2022 Brazilian National Examination for Medical Degree Revalidation (Revalida) and its potential as a tool for providing feedback on the examination's quality. Two independent physicians entered all examination questions into ChatGPT-4 and compared their responses to the test solutions, determining whether they were adequate, inadequate, or indeterminate. Consensus was used to resolve disagreements. The study also used statistical analysis to evaluate performance across medical themes and to eliminate queries.

In the Revalida examination, ChatGPT-4 correctly answered 71 (87.7%) of the questions and mistakenly answered 10 (12.3%). The proportion of correct responses did not change statistically significantly across medical themes. However, in nullified questions, the model had a lower accuracy of 71.4%, and there was no statistical difference between the non-nullified and nullified groups. The reliance on the judgments of only two independent physicians to evaluate the accuracy of ChatGPT-4 is a potential weakness of this study. This raises the likelihood of subjective bias in their evaluations. Furthermore, the study does not provide extensive information on the criteria used to categorize the model's replies as adequate, inadequate, or uncertain, which may impair the evaluation's credibility.

Furthermore, the study does not provide extensive information on the criteria used to categorize the model's replies as adequate, inadequate, or uncertain, which may impair the evaluation's credibility. Furthermore, the study does not investigate the reasons for ChatGPT-4's wrong answers, which could have provided useful insights for enhancing the model's performance. Furthermore, the study does not address the potential constraints or obstacles of evaluating a medical examination utilizing a broad language model like ChatGPT-4. Overall, while the study gives some insights into ChatGPT-4's competence in answering the Revalida examination, the study's small number of evaluators and insufficient information on evaluation criteria are significant weaknesses. More studies with a larger and more diverse sample are needed. Modern approaches and a large training set are needed to remove bias and errors from chatbots^2,3^. This is due to the possibility of issues arising when relying solely on a huge data source. Employing chatbots poses ethical questions since it could lead to unforeseen or undesirable effects. To prevent the dissemination of harmful ideas and incorrect information, ethical controls and restrictions must be put in place as artificial intelligence language models advance^4^.
